# Hiatus hernia with gastric volvulus and duodenum component: a case report

**DOI:** 10.1093/jscr/rjab548

**Published:** 2021-12-11

**Authors:** F Y Gimenez, L A R Takahashi, G G Oliveira, J Y Myaki, M E Inaba, F P A Feitosa, D Adão

**Affiliations:** General Surgery, Federal University of São Paulo, Durval Clemente Street, São Paulo, Brazil; General Surgery, Federal University of São Paulo, Durval Clemente Street, São Paulo, Brazil; General Surgery, Federal University of São Paulo, Durval Clemente Street, São Paulo, Brazil; General Surgery, Federal University of São Paulo, Durval Clemente Street, São Paulo, Brazil; General Surgery, Federal University of São Paulo, Durval Clemente Street, São Paulo, Brazil; General Surgery, Federal University of São Paulo, Durval Clemente Street, São Paulo, Brazil; General Surgery, Federal University of São Paulo, Durval Clemente Street, São Paulo, Brazil

## Abstract

Gastric volvulus is a rare condition defined as an abnormal stomach rotation around its axis, which usually presents in children under a year or in adults in their fifth decade. Cases over 70-year-old are rare and only 30% of cases of this disease present with mesenteric-axial rotation of the stomach.

In this article, we report a rare case of mesenteroaxial gastric volvulus associated with hiatal hernia, in an 88-year-old woman, who presented to the Emergency Department of our institution with bowel obstruction symptoms.

The diagnosis could be difficult due to the rarity of the pathology, the patient's age outside the expected incidence age range and the less common mesenteroaxial presentation.

This report highlights the importance of the differential diagnosis of gastric volvulus as a cause of intestinal obstruction.

## INTRODUCTION

Acute gastric volvulus is a rare condition, defined as an abnormal rotation of the stomach on its axis. Incidence varies between children and adults, with rare cases in adults over 70 years of age. Due to the risk of strangulation, the outcome can present as necrosis, perforation and hypovolemic shock, with a mortality rate reaching 30 to 50%, requiring early diagnosis and approach [1, 2].

We present a case report of an 88-year-old patient admitted to the Emergency Room of Hospital São Paulo with an unusual variation of the gastric volvulus, the mesenteroaxial, which corresponds to 30% of the cases.

The information presented in this study was obtained through a review of the medical record, interview with the patient and photographic record of the diagnostic methods to which the patient was submitted.

All team members take responsibility that attempts have been made to contact the family for signed consent. Unfortunately, contact with the patient’s family was not possible. And the patient died at the end of 2021 from complications related to COVID 19.

## PRESENTATION OF CASE

An 88-year-old female was admitted to the Emergency Room of Hospital São Paulo, with complains of nausea and vomiting for 3 days. Her medical background was unremarkable, except for Alzheimer’s Disease managed with valerian. She denied any surgical history.

**Figure 1:**
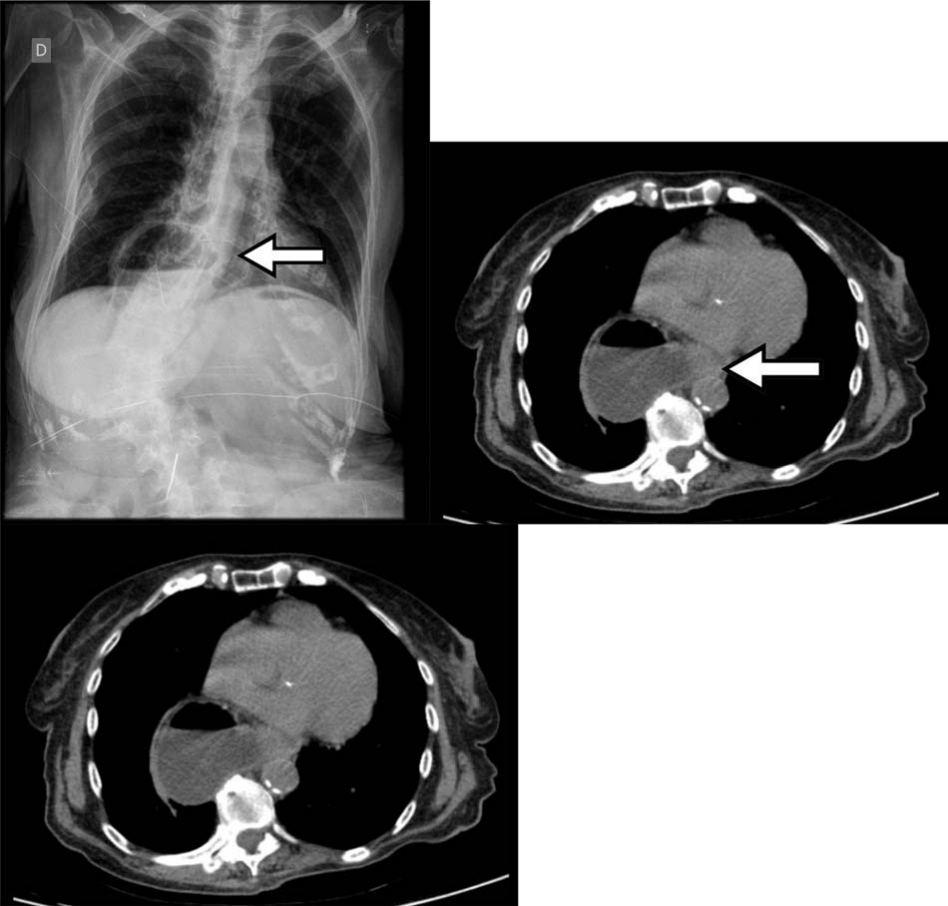
Chest X-ray with gas distension and the air-fluid level above the diaphragmatic domes and contrast-enhanced abdominal CT showing gastric volvulus in hiatal hernia.

Upon initial physical examination, the patient presented regular general condition with stable vital signs, flaccid abdomen without peritonitis signs, masses or visceromegaly. The rectal examination contained hard stools and no signs of bleeding. Due to the clinical presentation suggestive of upper gastrointestinal tract obstruction, laboratory tests were requested, which showed abnormal renal function (KDIGO 2). The abdominal radiography in three views showed air-fluid levels located in the epigastric region, corresponding to the distended stomach (Fig. 1). Abdominal tomography showed gastric hernia containing the antropyloric junction, which was located above the gastroesophageal junction, therefore, mesenteroaxial gastric volvulus.

Upper digestive endoscopy was performed in the operating room as a primary attempt of detortion, as well as to analyze the chronicity of the condition and signs of ischemia. Due to the failure of endoscopic detortion and intestinal subocclusion observed during the procedure, the patient underwent surgery shortly after endoscopy. Intraoperatively, the gastric antrum contained in the diaphragmatic hernia was visualized, along with several adhesions that hinder mechanical traction. Considering this condition and the absence of mucosal distress, an intestinal bypass was chosen as a form of treatment. An isoperistaltic gastroenteroanastomosis was performed between the gastric body and the duodenal loop, with a linear stapler, 30 cm from the angle of Treitz.

During the immediate postoperative period at Post Anesthetic Recovery, the patient used an oxygen catheter at 1 L/min, and an open nasogastric tube, remaining in fasting, showing acute worsening of renal function (KDIGO 3). On the seventh postoperative day, the patient required intensive care due to melena with hematimetric drop and decreased level of consciousness. So a new endoscopy was performed, which demonstrated a shallow ulcer with fibrin (Forrest 3) in the gastroenteric anastomosis, being a probable cause of the melena and not requiring any endoscopic treatment.

In the intensive care unit, the hydroelectrolyte disturbances and blood loss were managed accordingly, and an oral diet was initiated.

The patient was discharged after 16 days of hospitalization, with considerable improvement in baseline status. She returned to the postoperative outpatient clinic uneventfully, accepting the oral diet well, without new episodes of gastrointestinal tract bleeding.

## DISCUSSION

The primary form of volvulus occurs in the absence of diaphragmatic defects or intra-abdominal malformations, being correlated with alterations in the fixation of the stomach to its surroundings. The secondary form, which is more frequent (75%), occurs due to changes in gastric anatomy or functionality or anomalies adjacent to the organ [2].

Regarding the anatomical classification, the gastric volvulus can be organoaxial or mesenteroaxial, according to the rotation of the organ. In 60% of cases, rotation is organoaxial, characterized by rotation about the longitudinal axis, with the fixation points being the esophagogastric junction and the pylorus [2, 4]. In 30% of cases, rotation occurs mesenteroaxial, characterized by rotation about the transverse axis, overlapping antrum and pylorus under the esophagogastric junction. In 12% of cases, the two types of rotation coexist [2, 3].

Acute conditions are accompanied by pain in the upper abdomen or lower chest, associated with nausea or vomiting. These symptoms, associated with the inability to progress the nasogastric tube, make up Borchdat’s triad, present in 70% of cases [2, 3]. Hematemesis can occur as a result of mucosal ischemia associated with vomiting. Complications include ulceration, perforation, pancreatic necrosis and omentum avulsion [2].

In this case, the diagnosis could be difficult due to the rarity of the pathology itself, associated with the patient’s age outside the expected incidence range, as well as the less usual mesenteroaxial presentation.

In suspected gastric volvulus, the diagnostic investigation should start with a chest and total abdomen X-ray. If any diagnostic doubt, computed tomography should be made, as it is also capable of showing abnormalities associated with secondary volvulus [5]. Among tomographic findings, migration of the pylorus and narrowing of the hernia ring is most sensitive and specific for diagnosis [6, 7].

Despite being less accurate than tomography, upper digestive endoscopy has the advantage of evaluating the degree of ischemia of the stomach, in addition to being a therapeutic possibility for many cases, through the alpha loop maneuver for devolution [8].

The choice of the best surgical technique for the treatment of gastric volvulus depends on each case, mainly taking into account factors such as the etiology and chronicity of the volvulus.

Factors such as presence of a high obstruction syndrome of the gastrointestinal tract and the chronicity of the condition, in addition to the patient’s age and high surgical risk, were decisive for choosing this surgical technique. Thus, a smaller surgical procedure was prioritized, aiming at a lower rate of possible postoperative complications; this approach showed satisfactory resolution of the condition.

## CONCLUSIONS

This report highlights the importance of the differential diagnosis of gastric volvulus as a cause of intestinal obstruction.

## AUTHOR CONTRIBUTIONS

F.Y.G. and L.A.R.T. worked on article formatting and writing, G.G.O. and F.P.A.F. on bibliographic research, J.Y.M. on medical record search, M.E.I. on medical record search and D.A. worked on translation and final proof reading.

## CONSENT TO PUBLISH

The report had the consent form for publication of the case obtained by the patient’s family member.

## DATA AVAILABILITY

Only the data contained in this manuscript (article) will be available. If any reader wants to see the complete bank, through an appropriate request to the corresponding author, the author can send the requested database.

## COMPETING INTERESTS

The authors declare that they have no competing interests.
